# Negative Symptoms of Schizophrenia and Dopaminergic Transmission: Translational Models and Perspectives Opened by iPSC Techniques

**DOI:** 10.3389/fnins.2020.00632

**Published:** 2020-06-18

**Authors:** Ginetta Collo, Armida Mucci, Giulia M. Giordano, Emilio Merlo Pich, Silvana Galderisi

**Affiliations:** ^1^Department of Molecular and Translational Medicine, University of Brescia, Brescia, Italy; ^2^Department of Psychiatry, University of Campania “Luigi Vanvitelli”, Naples, Italy; ^3^Research & Development, Alfasigma Schweiz, Zofingen, Switzerland; ^4^Department of Brain Sciences, Imperial College London, London, United Kingdom

**Keywords:** schizophrenia, negative symptoms, dopamine, animal models, iPSC

## Abstract

Negative symptoms (NS) represent a heterogeneous dimension of schizophrenia (SCZ), associated with a poor functional outcome. A dysregulated dopamine (DA) system, including a reduced D1 receptor activation in the prefrontal cortex, DA hypoactivity in the caudate and alterations in D3 receptor activity, seems to contribute to the pathogenesis of NS. However, failure to take into account the NS heterogeneity has slowed down progress in research on their neurobiological correlates and discoveries of new effective treatments. A better neurobiological characterization of NS is needed, and this requires objective quantification of their features that can be applied in translational models, such as animal models and human inducible pluripotent stem cells (iPSC). In this review we summarize the evidence for dopaminergic alterations relevant to NS in translational animal models focusing on dysfunctional motivation, a core aspect of NS. Among others, experiments on mutant rodents with an overexpression of DA D2 or D3 receptors and the dopamine deficient mice are discussed. In the second part we summarize the findings from recent studies using iPSC to model the pathogenesis of SCZ. By retaining the genetic background of risk genetic variants, iPSC offer the possibility to study the effect of de novo mutations or inherited polymorphisms from subgroups of patients and their response to drugs, adding an important tool for personalized psychiatry. Given the key role of DA in NS, we focus on findings of iPSC-derived DA neurons. Since implementation of iPSC-derived neurons to study the neurobiology of SCZ is a relatively recent acquisition, the available data are limited. We highlight some methodological aspects of relevance in the interpretation of *in vitro* testing results, including limitations and strengths, offering a critical viewpoint for the implementation of future pharmacological studies aimed to the discovery and characterization of novel treatments for NS.

## Introduction

### Schizophrenia Dimensions and Subtypes

Schizophrenia (SCZ) is often a chronic, disabling mental disorder that affects about 1% of the world’s population. Pharmacological treatments have proven effective but have not substantially improved the functional outcome for the majority of people with SCZ, as they are not effective on dimensions which account for most of the functional impairment of these people (Fleischhacker et al., 2014; Galderisi et al., 2014, 2016, 2018b). Several observations concerning heterogeneity of risk factors, clinical picture, course, response to treatment, biological correlates, and functional outcome suggest heterogeneity of pathophysiological mechanisms (Kirkpatrick et al., 2001, 2017; Kirkpatrick and Galderisi, 2008).

Both dimensional and categorical approaches have been adopted to reduce the heterogeneity of the syndrome. The dimensions relevant to the phenomenology of primary psychotic disorders, most commonly reported by factor analytic studies (Liddle, 1987; Peralta and Cuesta, 2001; Rosenman et al., 2003; Demjaha et al., 2009), include: the positive (delusions and hallucinations), disorganization (including formal thought disorder, inappropriate affect, and disorganized behavior) and negative dimensions. However, the number of psychopathological dimensions and the symptoms included in each of them are still controversial (Peralta and Cuesta, 2001; Rosenman et al., 2003; Demjaha et al., 2009).

In particular, the negative symptom (NS) dimension is not unitary and can be subdivided in Avolition-apathy, including avolition, anhedonia and asociality, and Expressive deficit, including blunted affect and alogia, or in five distinct domains: avolition, anhedonia, asociality, blunted affect, and alogia (Kirkpatrick et al., 2006, 2011, 2017; Galderisi et al., 2013, 2018a; Mucci et al., 2015b, 2019; Ahmed et al., 2019; Strauss et al., 2019a, b). Secondary NS (i.e., those symptoms secondary to psychosis, depression, extrapyramidal side effects of antipsychotic drugs or environmental deprivation) represent a further source of heterogeneity. Recent meta-analyses have shown that available antipsychotic (AP) drugs, including clozapine, are not effective in the treatment of NS (Fusar-Poli et al., 2015). None of the included studies have distinguished between primary and secondary NS (Leucht et al., 2017). Only one manufacturer sponsored study showed the efficacy of cariprazine – a dopamine D3 partial agonist – on NS (Nemeth et al., 2017).

As to categorical approaches, two SCZ subtypes are still regarded as deserving investigation, though not included in classification systems: the Deficit Schizophrenia and the Treatment Resistant Schizophrenia.

Deficit Schizophrenia is characterized by the presence of primary and enduring NS; it shows different risk factors, signs and symptoms, course, response to treatment, and functional outcome, with respect to the non-deficit subtype of SCZ (Kirkpatrick et al., 2001, 2017; Kirkpatrick and Galderisi, 2008; Mucci et al., 2017). Subjects with Deficit Schizophrenia, as compared with those without the deficit features, have specific risk factors (i.e., male gender and summer birth), worse early premorbid social and academic adjustment, poorer general cognitive abilities (as assessed by intelligence quotient), more neurological soft signs and poorer functional outcome and response to treatment (Kirkpatrick et al., 2001, 2017; Galderisi et al., 2002; Kirkpatrick and Galderisi, 2008; Peralta et al., 2014; Bucci et al., 2016; Mucci et al., 2017). Treatment Resistant Schizophrenia (25–35% of subjects with SCZ) is identified *post hoc* after several attempts to treat the subject and might be related to different pathophysiological mechanisms with respect to treatment responsive SCZ. Thus, the identification of treatment-resistant cases might be useful to reduce heterogeneity of hypothesized pathophysiological mechanisms (Howes and Kapur, 2014; Howes et al., 2017a).

## Main Hypotheses on Pathophysiological Mechanisms of Schizophrenia

The Dopamine (DA) hypothesis is rooted in the knowledge of the role of DA transmission in controlling behavior across species, in the association between DA D2–D3 receptor blockade and clinical efficacy of AP on positive symptoms as well as in the psychotogenic and in the psychotomimetic effects produced by DA enhancing substances.

However, about one third of subjects with SCZ does not respond to AP as reported above; furthermore, cognitive deficits and primary NS are not improved by AP, while they predate the onset of psychosis and are probably related to neurodevelopmental abnormalities, which might represent core aspects of the pathophysiology of SCZ.

A complex hypothesis attempts to reconcile these observations, in which a subcortical DA excess (with enhanced transmission at the postsynaptic D2–D3 receptors) is associated to prefrontal DA hypofunction (at the D1 receptor, which is the most diffuse receptor type in the cortex). Cortical hypoDA function has been related to NS and cognitive deficits (Weinberger, 1987; Howes and Kapur, 2009; Laruelle, 2014; Howes et al., 2017b). However, a meta-analysis of imaging correlates of psychopathological dimensions has not confirmed a relationship between dorsolateral prefrontal cortex hypofunction and NS (Goghari et al., 2010). The “revised” DA hypothesis of SCZ (Howes and Kapur, 2009) is based on the association between severity of psychosis and increased DA transmission in the dorsomedial, associative striatum more than in the ventral striatum/nucleus accumbens. This is at odds with the first formulation of the hypothesis that highlighted the role of the ventral striatum or limbic striatum for psychosis and of the dorsal striatum for extrapyramidal side effects (Laruelle, 2014). The SCZ pathophysiology would include a reduced DA transmission in the nucleus accumbens, related to NS; an increased DA transmission in the associative striatum related to psychosis, and a reduced DA transmission in the neocortex related to cognitive deficits (Howes and Kapur, 2009; Laruelle, 2014; Howes et al., 2017b).

Dopamine transmission alterations might also derive from a cortical glutamatergic dysfunction involving the NMDA receptor (NMDA-R, which is modulated in opposite directions by D1 and D2 receptors). Glutamatergic signaling deficits are regarded as one of the earliest neurodevelopmental abnormalities associated with SCZ, with primary deficits in NMDA-R signaling, particularly in layer 3 pyramidal neurons in prefrontal cortex (Jardri et al., 2016). These deficits lead to an excitatory/inhibitory imbalance and are thought to underlie working memory deficits; they seem to impair recurrent excitation and thus the maintenance of information in the working memory. However, the mechanisms by which excitatory/inhibitory imbalance at the cellular level might relate to clinical symptoms remain unclear.

Finally, in consideration of the well-known role in motivation and incentive behavior, the DA hypothesis provides a reasonable neurobiological substrate to the salience hypothesis (Howes and Kapur, 2009; Howes and Nour, 2016; Howes et al., 2017b) and, at the same time a conceptual framework that links risk factors, including pregnancy, obstetric complications, early life stress, trauma, drug use, and gene variants, that are known to increase presynaptic striatal DA transmission.

### Dopaminergic Dysfunctions Relevant to Negative Symptoms

As outlined above, NS are heterogeneous. Investigation of neurobiological correlates of primary vs. secondary NS and motivation-related vs. expression-related symptoms has not been systematic (Kirkpatrick et al., 2017; Galderisi et al., 2018a). However, some hypotheses can be formulated based on the existing literature. In particular, motivation impairment, underlying avolition, asociality, and possibly anhedonia, can derive from different DA pathophysiological mechanisms, involving either the salience or the positive-valence system (Galderisi et al., 2018a). The pattern of motivational deficit found more often in SCZ includes a reduced anticipation of reward (which is still discussed as a possible correlate of depression), enhanced effort discounting and reduced valuation of action, in the presence of a preserved hedonic response (Barch et al., 2016; Galderisi et al., 2018a). Motivation-related deficits in SCZ seem to recognize mechanisms different from those more often observed in depression, in which the poor motivation is associated with a reduced sensitivity to reinforcers and pleasure. Several brain imaging studies found in SCZ alterations of the DA-dependent response of the ventral striatum to reward anticipation and an association with NS (Radua et al., 2015). However, in other studies, the same alterations were found to correlate with positive symptoms, and not with NS (Nielsen et al., 2012), and discrepant findings (i.e., no significant deviation of the DA-dependent response in the ventral striatum) were observed in subjects treated with second generation antipsychotics (SGA) (Juckel et al., 2006; Schlagenhauf et al., 2008; Mucci et al., 2015a). Furthermore, studies in which an association between reduced ventral striatum response and NS was found, generally used the negative subscale total of the Positive and Negative Syndrome Scale (PANSS), which includes ratings of cognitive deficits and a poorly sensitive assessment of Avolition-apathy. When a better assessment of Avolition-apathy was achieved with the Schedule for Deficit Syndrome (SDS), subjects with SCZ and high Avolition-apathy severity showed reduced fMRI BOLD response of associative striatum and normal response of the nucleus accumbens to reward anticipation, when compared to both healthy controls and subjects with SCZ and low Avolition-apathy severity; the same pattern was observed in Deficit Schizophrenia subjects. The reduced imaging bold signal in associative striatum also correlated with reduced real-life motivation (Mucci et al., 2015a). These subjects, characterized by high Avolition-apathy severity, showed also abnormalities of structural and functional connectivity in the motivation-related circuits (Amodio et al., 2018; Giordano et al., 2018). Reduced associative striatum activation during a devaluation task was observed in subjects with SCZ and was found to be associated with the Scale for the Assessment of Negative symptoms (SANS) Avolition-apathy subscale (Morris et al., 2015).

### Impaired Motivation Due to Reduced DA Transmission in the Limbic or Associative Striatum: Translational Animal Models

Because of the importance of effort-related dysfunctions in SCZ, animal tests of effort-based decision-making have recently been used to develop formal models of motivational symptoms. In rodents, disconnection studies have shown that a distributed circuit is involved in effort discounting: nucleus accumbens, amygdala (basolateral), prefrontal and anterior cingulate cortex, and basolateral pallidal neurons. D1/D2 antagonists or DA depletion in nucleus accumbens bias behavior toward effort discounting, leaving intact the hedonic reaction and learning of reward-stimuli associations; the same is obtained by inactivation of nucleus accumbens core neurons by local GABA_A/B_ receptor blockade (Salamone et al., 2016b). Experiments involving a progressive effort-reward ratio task showed high variability of performance (i.e., of engagement on progressive higher effort to obtain valued outcomes, up to the break point when effort exceeds outcome); in fact, some rats engaged in motivated lever press behavior very little (low responders), while others did much more (high responders), suggesting differential DA system involvement. The D1-dependent signal transduction marker pDARPP-32(Thr34) (i.e., DARPP-32 phosphorylated at the threonine 34 residue) was found significantly more expressed in the nucleus accumbens core in high responders compared to low responders (Salamone et al., 2016b). In addition, several neurotransmitters interact with the DA signaling in order to regulate effort-related functions. For instance, DA D2R interacts with adenosine A_2A_ receptors to regulate effort discounting; due to this interaction, antagonists of A_2A_ receptors reverse DA D2R antagonism on effort discounting. Intra-NAcc injection of pilocarpine, a muscarinic agonist, interferes with DA transmission and produces effort discounting. Reduced selection of high effort choices in rodents can be induced by the administration of tetrabenazine (TBZ) which inhibits the vesicular monoamine transporter type 2 (VMAT-2). The inhibition of VMAT-2, encoded by Slc18a2, results in reduced vesicular storage and depletion of monoamines, and produces the same pattern of preference for low-effort option, without interfering with the devaluation test (which indicates integrity of outcome valuation) (Salamone et al., 2016a). The VMAT-2 inhibitor TBZ produces the low-effort bias without impairing the sensitivity to reinforcers (in a maze test when no barrier is used, treated rats will get the valued food in the correct arm), memory or orientation (Salamone et al., 2016a; Yohn et al., 2016). TBZ effects on motivation can be reverted by bupropion and by A_2A_ receptor antagonists (clinically used as antiparkinsonism agents). The A_2A_ receptors are co-localized on enkephalin-positive medium spiny neurons in both dorsal striatum and nucleus accumbens (Salamone et al., 2016a; Yohn et al., 2016). The models above seem related to motivational deficits relevant to depression (reverted by bupropion) and secondary NS related to AP (reverted by antiparkinsonism drugs).

Animal models of anhedonia and asociality have focused mainly on NMDA-R activity, using, for instance, NMDA-R antagonists such as phencyclidine (PCP), MK-801 and ketamine. The exposure of rats to PCP, an antagonist of NMDA-R, mimics cognitive deficits and NS. In particular, it has been observed that the anhedonic effect of the NMDA antagonisms appears at doses greater than those that produce other effects such as asociality and cognitive deficits and also induces neural pathology distinct from that observed in SCZ. In addition, this anhedonic effect does not contribute to disentangle the two aspects of anhedonia (anticipatory and consummatory) (Neill et al., 2014). In rodents, the acute and chronic administration of MK-801 led to social interaction deficits. The sub-chronic administration of ketamine induced NS-related behaviors, while findings concerning the acute administration, which induce rapid and transient psychotomimetic effects, have been less consistent (Lee and Zhou, 2019). The postnatal NR1 knockout mutant mice, a model which produces early postnatal inhibition of NMDA-R activity in corticolimbic GABAergic interneurons, contributes to the onset of SCZ-like abnormalities (novelty-induced increase in locomotion, mating and nest-building difficulties, anxiety and anhedonia-like behaviors) in adult rodents. These behavioral abnormalities are exacerbated by social isolation and associated with deficits of social memory, spatial working memory and prepulse inhibition (Belforte et al., 2010).

Animal models of poor motivation of SCZ are represented by the DA deficient mice induced, for example, with local neurotoxic injection with 6-OHDA in nucleus accumbens, mutant rodents with an overexpression of DA D2R (DR2-OE) or an overexpression of DA D3R (DR3-OE).

Accumbal DA deficient mice have no impairment in hedonic reactions but prefer low-effort options, showing no willingness to work to reach valued outcome (Salamone et al., 2016b). In these models reward valuation and motivation are separable processes and value computations seem to be intact. Motivation to work for large rewards can be restored improving DA transmission in the dorsomedial striatum (Palmiter, 2008).

Overexpression of DA D2R in striatal medium spiny neurons specifically induced by a viral vector strategy in adult mice leads to increased behavioral activation and effort expenditure (Trifilieff et al., 2013), while the same overexpression during development leads to the opposite effect with effort discounting (Ward, 2016). Mutants with constitutive DR2-OE show effort discounting, without reduced sensitivity for rewarding outcome (if no effort is required, they prefer the valued outcome). However, these rodents also show an impaired valuation of response outcome, as if a disconnection of value and action were present. Overall, the deficit in effort-related choices in constitutive DR2-OE mice seem to be due to reduced sensitivity to the value of different response options. Upregulation of D3 receptors in constitutive DR3-OE was associated with a downregulation of striatal D1R and led to an impairment of the motivation and incentive behavior, with preserved basic behaviors and cognitive functions (Simpson et al., 2014). However, given the pre- and postsynaptic expression of D3 receptors in different limbic structures, these data cannot be conclusive, pointing to the importance to test the effects of pharmacological agents acting on D3R receptors on motivational impairment (Neill et al., 2016; Sokoloff and Le Foll, 2017), as described in the following paragraph.

Overall, these animal models have improved knowledge on motivation impairment that characterize NS, but they did not significantly contribute to clarify pathophysiological mechanisms underlying the other NS dimensions (i.e., alogia) and their interrelationships.

### The Clinical Relevance of D3 Receptor in Targeting Negative Symptoms

Recent advances in the studies of D3 receptor structure, function and properties have provided a boost in the search and discovery of D3 selective ligands (Platania et al., 2012; Leggio et al., 2016; Maramai et al., 2016; Sokoloff and Le Foll, 2017; Torrisi et al., 2017). These receptors are predominantly located in areas that are relevant to psychotic symptoms, such as ventral mesencephalon, striatum, thalamus, hippocampus, and frontal cortex (Leggio et al., 2016; Maramai et al., 2016; Sokoloff and Le Foll, 2017).

D3 receptors have a higher affinity for DA than D2 receptors; their ligand binding does not lead to the classical D2 receptor subtype side effects (e.g., extrapyramidal symptoms) and in preclinical models results in improvement of cognitive impairment and NS (Leggio et al., 2016; Maramai et al., 2016; Sokoloff and Le Foll, 2017). As reported above, the overexpression of D3 receptor in striatum disrupts motivation, suggesting that changes in D3 receptor may be involved in NS (Simpson et al., 2014). On the contrary, null mutation (KO) of D3 receptors or administration of D3 receptor antagonists are associated with the attenuation of enhanced motivation produced by the excessive DA release induced by psychostimulants (Song et al., 2012), suggesting a complex interplay between presynaptic and postsynaptic D3 receptors (Merlo and Collo, 2015). Therefore, D3 partial agonist/antagonism would be a valuable approach for the treatment of NS.

Most AP, both first and second generation, do not display selectivity for D3 over D2 receptors, but few compounds, including aripiprazole, blonanserin, and cariprazine, show some D3 selectivity (Leggio et al., 2016). Among these AP, only cariprazine, a DA D3-preferring D3/D2 receptor partial agonist and serotonin 5-HT1A receptor partial agonist, showed an impact on NS. In animal models of rats exposed to chronic mild stress, cariprazine attenuated the decrease in sucrose intake, which represents a model of anhedonia (Leggio et al., 2016). To date, in human, cariprazine is the only AP that has proven more effective than other SGA (i.e., risperidone) in improving NS in subjects with SCZ with predominant and persistent NS (Nemeth et al., 2017; Fleischhacker et al., 2019). Compared to cariprazine, aripiprazole, which is a D2/D3 and 5-HT1A partial agonist, has a lower affinity at D3 receptor. In rodents, aripiprazole does not lead to improvement in social behaviors (Leggio et al., 2016). Further effort is needed in order to improve our knowledge in the involvement of D3R in NS for development of effective treatments.

## Inducible Pluripotent Stem Cells

Inducible pluripotent stem cells (iPSC) from human donors are a relatively new tool in translational research for disease modeling, as well as for the discovery of novel pharmacologic therapeutics. Different robust protocols have been developed in order to generate iPSC from somatic cells of human donors that are reprogrammed into a pluripotent state through the forced expression of a set of transcription factors (Oct3/4, Sox2, Klf4, c-Myc) that affects their epigenetic state (Takahashi et al., 2007; Aasen et al., 2008; Staerk et al., 2010; Petit et al., 2012). Differentiating human iPSC into specific central nervous system (CNS) phenotypes (e.g., neurons, glia cells) with acceptable fidelity compared to *in vivo* brain neurons, offers the possibility to generate what has been called “disease models in a dish” (Inoue and Yamanaka, 2011; Marchetto et al., 2011; Penney et al., 2019). One critical aspect of iPSC-derived neurons is that the genome of the donor is represented in each iPSC clone, while the epigenetic features of the tissue of origin are almost completely erased. When a standardized and highly controlled process of differentiation is applied *in vitro*, the resulting difference of iPSC-derived neurons from healthy donors should mainly depend on the original genetic makeup related to the disorders, allowing the assessment of the modulatory effects of genetic mutations on cellular functions (Deflorio et al., 2017; Penney et al., 2019). Given the role that DA system plays in NS, in this review we focus on the evidence supporting the use of human DA neurons derived from iPSC as a potential translational approach for a better understanding of the molecular and cellular mechanisms associated to SCZ with persistent and primary NS. Since implementing iPSC-derived neurons to study the neurobiology of psychiatric disease is a relatively recent acquisition, the available data are limited. An initial translational approach was proposed using iPSC-derived forebrain neurons from donors with SCZ (reviewed in Soliman et al., 2017; Balan et al., 2019), while pharmacological studies using neuroactive drugs known to produce psychotomimetic effects were performed in iPSC-derived mesencephalic DA neurons (Cavalleri et al., 2018). In the following paragraphs these findings will be summarized (see also Table 1) and some research about future directions will be proposed.

**TABLE 1:** Pharmacological studies using iPSC derived neurons from subjects with schizophrenia.

**Donors**	**Neuronal types**	**Differentiation time (days)**	**Relevant phenotypic changes**	**Effective pharmacological agents**	**Non-effective pharmacological agents**	**References**
Schizophrenia patients from high risk families: one childhood onset schizophrenia; two affected siblings (one schizophrenia and one schizoaffective disorder); one adult schizophrenia patient	Glutamatergic (70%), GABAergic (30%), dopaminergic less than 10%	1–3 months	Reduced dendritic number, neuronal connectivity and dendritic spines. Lower expression of glutamate receptors subunits GRIK1, GRIN2A, GRM7. Defective Wnt (e.g., decreased WNT7A) and cAMP signaling (e.g., increased PDE4D, PDE7B)	Loxapine (10 μM) increases neuronal connectivity	Clozapine (5 μM) Olanzapine (1 μM) Risperidone (10 μM) Thioridazine (5 μM)	Brennand et al., 2011
Members of a DISK 1 mutation family: one schizophrenia patient, one patient with ajor depression and two unaffected subjects. Isogenic iPSc lines	Glutamatergic (90%), very few GABAergic and dopaminergic	Up to 6 weeks	Reduced synaptic vesicle protein SV2 density, reduced vesicle release (FM1-43) and spontaneous ESP. Increase expression of SYN2 SYN3, SYP, SYNPR, NRXN1, VAMP2. Increase expression of transcription factor MEF2C.	Rolipram, reverses synaptic deficit		Wen et al., 2014, 2017
Monozygotic twin patients with treatment resistant schizophrenia, one responder to clozapine and one non-responder	Tuj1 positive neurons	2 weeks following Ngn2 overexpression	Reduced homophilic cell adhesion molecules (e.g., *CDH8*, *DSC*) protocadherin genes.	Clozapine (1 μM), differential gene expression between responder and not responder to clinical treatment		Nakazawa et al., 2017
Nine patients with treatment resistant schizophrenia and nine healthy donors	GABAergic cortical interneurons	6–8 weeks	Reduced ND2 and ND4L NADH dehydrogenases (complex I) Decrease mitochondrial function (maximal respiration and reserve capacity) Increased oxidative stress	Acetyl-l-carnitine, significant increases maximal respiration and reserve capacity Ameliorate arborization deficits	Omega-3 fatty acids, coenzyme Q10, N-acetyl cysteine, α-tocopherol	Ni et al., 2019

### The Target Tissue: Midbrain DA Neurons and Their Embryologic Development

In adult brain the midbrain DA (mDA) neurons are mainly localized in the ventral part of the mesencephalon, harbored within the substantia nigra pars compacta (SN, A9) and, more medially, into the ventral tegmental area (VTA, A10). The nigrostriatal DA projections originated from the SN preferentially innervate the dorsolateral part of the striatum through the nigrostriatal pathways, while the mesolimbic pathway and mesocortical pathway originated from the VTA innervate the ventral part of the striatum and the infralimbic prefrontal cortex, respectively. During development, mDA neurons arise from the midbrain-hindbrain junction sending their terminals to various regions of the developing forebrain. In this period, at the midbrain-hindbrain boundary, DA neuron precursors express the transcription factors OTX2, known to play a critical role during the differentiation (Maxwell and Li, 2005). Later, DA cells develop in the ventral midbrain mostly due to the upregulation and release of sonic hedgehog (SHH) from the notochord, and Fgf8 from the isthmus. Other factors implicated in early development of mDA neurons include Wnt1, En1, En2, Nurr1, Pitx3, Lmx1B, Foxa2, and Pax5 (Maxwell and Li, 2005). Ultimately, maturation of mDA neurons is marked by the expression of tyrosine hydroxylase (TH) and dopamine transporter (DAT) in dendrites and soma, VMAT and SYN1 expression in the vesicle of synaptic terminals, the evidence of functional release and uptake of DA and the repetitive burst firing, a signature of electrophysiological DA neuron phenotype (Weihe et al., 2006).

### iPSC Differentiation Into Human DA Neurons

Several protocols capable to differentiate neurons with developmental trajectory resembling that of ventral mDA neurons have been described (Chambers et al., 2009; Kriks et al., 2011; Fedele et al., 2017; Marton and Ioannidis, 2019). Accordingly, ventral mDA neurons are identified by the co-expression of developmental markers. At first, iPSC are driven towards a phenotype that is equivalent to the progenitor status, defined using appropriate combinations of markers for forebrain/midbrain, i.e., OTX2, FOXA2, and LMX1A expression. Intermediate zone-like progenitors are then identified by FOXA2 co-expression with midbrain markers NURR1 and engrailed1 (EN1). Mantle zone neurons are identified by co-expression of FOXA2 with TH, the key enzyme for production of DA, and by the ventral mDA neuronal identifier PITX3. Terminal differentiation has further supported by the co-expression of functional markers such as DAT, D3R, GluR1/2, NR2A/B (Kriks et al., 2011; Cavalleri et al., 2018; Collo et al., 2018), by neurochemical properties, i.e., release and uptake of DA, and by electrophysiological signature, e.g., the burst firing typical of native DA neurons of the mammal ventral mesencephalon (Devine et al., 2011; Kriks et al., 2011; Collo et al., 2018). Recent transplantation experiments in rodents and monkeys using human iPSC derived DA precursors from healthy and parkinsonian donors have demonstrated their capacity to survive and integrate in the host tissue, showing newly innervation to striatum after neurotoxic lesions and improving defective motor activity via functional integration (Hallett et al., 2015; Kikuchi et al., 2017a, b).

### Fidelity of iPSC Derived Neurons *in vitro* Models: 2D Culture and 3D Culture-Organoids

In vitro iPSC differentiation has been preferentially performed using 2D cultures protocols, but recent technological improvements allow also 3D cultures, as exemplified by the successful development of human brain organoids (Lancaster et al., 2013; Arlotta, 2018).

The cellular phenotypes obtained by these methodologies reach a variable level of identity between iPSC-derived DA neurons vs. the mDA neurons in the brain when probed using transcriptomics and electrophysiological profiling. For example, using iPSC-derived DA neurons differentiated in 2D cultures up to 50 days, global differences in gene expression were found, in particular for genes related to the level of neuronal maturation (Xia et al., 2016). By using human midbrain-like organoids cultured up to 2 months, these differences were much reduced (Jo et al., 2016); a comparison of the genes expressed by these midbrain-like organoids with genes expressed by human DA neurons grown in 2D cultures (Lin et al., 2016a) and with available data from human mDA neurons (GTEx Consortium, 2015) showed an overlap with the gene expression profile of prenatal midbrains. The major difference with the 2D cultures was the presence of markers for astroglia and oligodendrocytes usually detected at late stages or not detected at all in 2D cultures (Jo et al., 2016). An important contribution regarding the critical role of the culture heterogeneity, i.e., the relative amount of different cell types presents in the samples analyzed for transcriptomics, was recently provided by Sandor et al. (2017). Using a TH-tagged intracellular marker, the transcriptomic profiles of iPSC-derived DA neurons revealed high similarity to the profile of post-mortem human mDA neurons. When DA neurons from Parkinson’s disease patients carrying LRRK2 G2019S genetic variants were compared to control individuals, functional convergence of differentially expressed genes was observed. However, no functional convergence amongst differentially expressed genes was found when non-purified iPSC-derived DA neurons were used. These data indicate that cellular heterogeneity observed in iPSC-derived neuronal cultures, i.e., the presence of variable number of non-DA neurons and astrocytes, could be a major confounder in assessing transcriptomic profiles, possibly more relevant to the difference of individual genotypic background (Sandor et al., 2017). Moreover, iPSC-derived DA neurons possess electrophysiological and synaptic properties similar to those of native mDA neurons (Kriks et al., 2011; Hartfield et al., 2014; Fedele et al., 2017). These single-cell functional features are at the basis of the development of networks of actively firing neurons that can generate bursting activity typical of functional circuits assessed *in vivo*.

Two main approaches for growing human brain organoids were proposed. The first approach, exemplified by Quadrato et al. (2016), allows human iPSC to form a layer of stem cells, the neuroepithelium, from which neuronal precursors differentiate and self-organize into multiple brain-like sub-regions. The authors provided the gene expression profiling in over 80,000 individual cells isolated from 31 human brain organoids after 9 months in culture and they identified several neuronal types (GABAergic, glutamatergic and also DAergic) interconnected in circuits via mature synapses, visualized using electron-microscopy, and organized in sub-regions capable to detect and react to external stimuli (Quadrato et al., 2016). In the second approach, exemplified by Birey et al. (2017) and Xia et al. (2016), a series of signaling factors was used to control the patterning of neuroepithelium development so to define specific brain sub-regions, e.g., forebrain or hypothalamus. This technique is presently hampered by the limited understanding of the signaling factors required for leading the development of several specific sub-regions. Notwithstanding these limitations, both approaches have so far enriched our understanding of the development of human brain circuit.

Initial studies on midbrain DA-like neurons were performed on human organoids derived from embryonic stem cells (Jo et al., 2016) or from regionally patterned neuroepithelial stem cells (Simao et al., 2015; Monzel et al., 2017) after 50–120 days of culture. These multicellular organoids contained spatially organized groups of DA neurons expressing markers typically found *in vivo* in A9 mDA (e.g., GIRK2, DAT) or A10 mDA (e.g., Calcineurin), as well as anatomically mature synapses, synaptic vesicle trafficking, release of DA in response to depolarizing stimuli, polarized membrane potential (Vm = −70 mV), voltage-dependent potassium currents, spontaneous calcium transients, spontaneous electrophysiological peacemaker activity that was reactive to the D2/D3-preferential DA agonist quinpirole via D2/D3 autoreceptor stimulation (Simao et al., 2015; Jo et al., 2016; Monzel et al., 2017). In addition, in human organoid cultures but not in mouse organoids or 2D cultures, neuromelanin-like granules were found accumulating in DA neurons over time (Jo et al., 2016).

In conclusion, the current human iPSC differentiation protocols provide consistent degree of fidelity with the original *in vivo* DA neuron phenotypes, generally higher in 3D organoids vs. 2D cultures. The molecular and morphological phenotypes remind a late-fetal or early-infantile phenotype, probably better positioned for modeling developmental disorders occurring in children or adolescent individuals, e.g., autism or SCZ (Quadrato et al., 2016; Soliman et al., 2017), rather than disorders emerging later in life.

## Phenotype of Human IPSC-Derived Neurons From Donors With Schizophrenia

**FIGURE 1:**
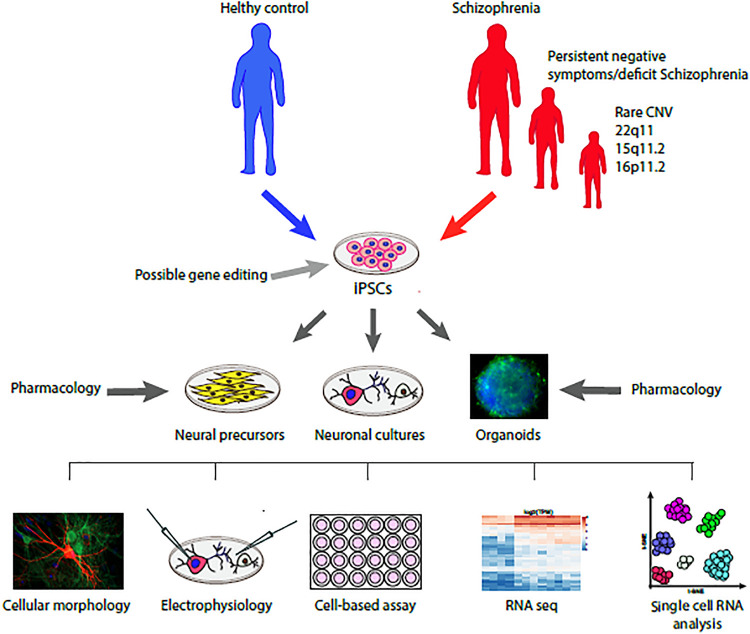
Schematic representation of the translational model to study the cellular and molecular phenotype of iPSC derived neurons from donors with schizophrenia compared with healthy controls and their response to pharmacological agents. Further evolution of this working model will include a precision medicine approach driven by patient stratification based on specific clinical descriptors (e.g., negative symptoms) or genetic liabilities (e.g., rare CNVs) associated to possible gene editing intervention.

In SCZ, the most common cellular pathology observed in post-mortem studies includes reduced dendritic complexity, reduced synaptic spine number and neuronal size of cortical neurons. This pathology was proposed to be due to a neurodevelopmental liability that results in a greater elimination of dendritic spine (“pruning”) during infancy and adolescence, but its molecular mechanisms are still unclear (Moyer et al., 2015). Intriguingly, the phenotypes obtained by most investigators using iPSC-derived neurons from donors with SCZ show reduced dendritic spine and reduced synaptic functions, hinting to a neurodevelopment-related impairment (Ahmad et al., 2018; Figure 1). Most of these data were obtained in cortical-like glutamatergic neurons, while only few articles were dedicated to study the DA neuron phenotype (Balan et al., 2019).

The first evidence of defective phenotype in iPSC-derived glutamatergic neurons from subjects with SCZ was published by Brennand et al. (2011). After 3 months in culture with astrocytes, glutamatergic cortical neurons from subjects with SCZ were morphologically different from those of healthy controls, displaying a reduced number of dendritic spines, reduced neuronal connectivity (as measured with rabies virus tracing assay), lower expression of some glutamate receptor subunits (GRIK1, GRM7, GRIN2A) and defective Wnt (e.g., decreased WNT7B) and cGMP/cAMP signaling (e.g., increased PDE4). These data are in line with the postmortem observation of reduced dendritic spines in cortical pyramidal neurons and reduced gray matter volume in SCZ, features that are not affected by AP or SGA treatments (Konopaske et al., 2014); instead, they were associated with NS (Roth et al., 2004). Defective dendritic and synaptic phenotypes of iPSC-derived neurons were confirmed by several laboratories (Ahmad et al., 2018; Balan et al., 2019). This defective phenotype may be related to down-regulation of key neurodevelopmental genes. For example, evidence of consistent down-regulation of NPTX2, a protein involved in excitatory synapse, was obtained when transcriptomics datasets from post-mortem brain and from iPSC-derived neurons were meta-analytically compared between SCZ and matched healthy subjects (Manchia et al., 2017). However, at present, no iPSC-derived neurons were generated from donors with SCZ with prevalent or persistent NS. Indirect information can be collected from studies on iPSC donated from treatment-resistant SCZ patients under current clozapine treatment (see Table 1), clozapine being used in subjects with prevalent NS (Brar et al., 1997).

Our knowledge of defective phenotypes of iPSC-derived DA neurons are based on the observations of Robicsek et al. (2013) reporting a delayed differentiation and maturation with reduced neurite length and number, low expression of TH and β3-tubulin and no DAT expression. In these cultures, glutamatergic neurons showed also a defective maturation, displaying reduced synaptic contacts. These results are in keeping with the defective development of cortical neurons and the prefrontal hypofunction of the corticolimbic DA system in SCZ patients (see paragraph 2). Subsequently, Hook et al. (2014) observed a relatively unaffected maturation of DA neurons, but reported an increased DA production and release, featuring the same key aspect of an overactive subcortical DA system observed in basal ganglia and ventral mesencephalon (Howes and Nour, 2016). Methodological differences between the two studies were highlighted by Hartley et al. (2015). Using a protocol that enhanced FOXA2 expression to drive the midbrain phenotype, they obtained DA neurons that did not display major differentiation and maturation defects. These data suggest a critical role of specific protocols, possibly favoring the development of different subpopulations of DA neurons with different functional phenotypes. However, this interpretation is still hypothetical given the limited amount of published data, the unchecked reproducibility of the procedures and the genetic heterogeneity of donors.

### iPSC-Derived Neurons in Genetically Profiled Subjects With Schziophrenia

Since iPSC retain the genetic characteristics of the donors and the heritability for SCZ is high, about 80% in twin studies (Hilker et al., 2018), it has been proposed that iPSC could reliably reproduce some critical aspect of the cellular biology associated to risk gene variants. A landmark meta-analysis, the Schizophrenia Working Group of the Psychiatric Genomics Consortium (2014) profiled common SNP variants from about 36 K subjects with SCZ and identified 128 common variant associations covering 108 independent loci. These loci include regions containing genes relevant for neurodevelopmental, synaptic function and plasticity, glutamatergic and DA neurotransmission, neuronal calcium and G-protein coupled receptor signaling and ion channels. A recent meta-analysis performed in about 67 K cases by the Psychiatric Genetics Consortium in Schizophrenia-3, confirmed the 108 independent loci and added additional 152 novel associations (O’Donovan, 2019). The strongest association was with complement C4 gene variants, proposed to be involved in the enhanced synaptic pruning observed during neurodevelopment in children and adolescents. Using an *in vitro* model featuring iPSC-derived microglia and cortical neurons from SCZ donors, Sellgren et al. (2019) demonstrate an increased rate of synapse engulfment and elimination. In this model, the effects on DA neurons were not studied.

Approximately 1.4% of the cases in genome-wide significant association are CNVs preferentially observed in 1q21.1, 2p16.3, 3q29, 7q11.2, 15q11.2, distal and proximal 16p11.2 and 22q11.2 (Marshall et al., 2017). The resulting syndromes generally affect young individuals and include comorbidities of SCZ, intellectual disabilities, autisms and epilepsy. Several recent studies profiled the phenotype of iPSC-derived neurons from donors with SCZ associated with CNVs, whose deficits were most likely produced by the genetic mutation. Among the neurons obtained from iPSC most were glutamatergic, few were GABAergic and none were DA neurons (Hoffmann et al., 2018). Delayed development was observed in iPSC-derived glutamatergic neurons from donors with 22q11 microdeletion (Pedrosa et al., 2011). More recently, RNA-seq analysis was used to investigate gene expression in iPSCs-neurons from donors with SCZ and autism spectrum disorders due to 22q11.2, showing downregulation of 753 genes, the majority being included in apoptosis, cell cycle, cell survival and MAPK signaling Gene Ontology, with a particular attention to the sub-network modulated by PRODH (Lin et al., 2016b). Studies on iPSC-derived neurons from subjects with 15q11.2 mutation also showed defective synaptic and dendritic development (Das et al., 2015). Intriguingly, different results were obtained with iPSC-derived neurons from subjects with 16p deletion, showing neuronal hypertrophy with increases in soma size and dendrite arborization, whereas 16p duplication iPSC derived neurons showed the opposite phenotype, with a reduction of dendritic arborization, especially in excitatory neurons (Deshpande et al., 2017).

A series of interesting studies were conducted using iPSC-derived forebrain neurons obtained from donors affected from a familial form of SCZ and major psychiatric disorders that co-segregate with a mutation of the disrupted-in-schizophrenia-1 (DISC1) gene (Wen et al., 2014; Ye et al., 2017; Wang et al., 2019). DISC1 is a scaffold protein acting as a key regulator of neuronal intracellular trafficking of vesicles and proteins, interacting also with DA D2 receptor (Su et al., 2014). In the first article deficits in synaptic number and synaptic vesicle release was observed in iPSC-derived neurons from carriers with a DISC1 frameshift mutations. The synaptic deficits were reverted by the correction operated via DISC1 gene editing in the isogenic cells (Wen et al., 2014). In a more recent article, using the same iPSC-derived neurons, these authors showed that the phenotypic synaptic impairments were related to the transcriptional dysregulation due to the defective interaction between the Activating Transcription Factor 4 (ATF4) and DISC1 (Wang et al., 2019). In another study, iPSC-derived neurons and organoids developed from the same DISC1 mutants were successfully used to investigate the role of the disrupted DISC1-Ndel1 complex on defective neurogenesis, a critical neurodevelopmental phenomenon in SCZ (Ye et al., 2017).

## Pharmacologic Studies in IPSC-Derived Neurons

As recently reviewed (Ahmad et al., 2018; Balan et al., 2019), the learning from iPSC derived neurons has a great potential in unraveling critical mechanisms for novel therapeutics for SCZ (Figure 1). However, so far, few studies have been dedicated to harness the potential of *in vitro* pharmacology in human iPSC-derived neurons. In Table 1 we summarize the studies using pharmacological approaches in iPSC-derived neurons of relevance for SCZ research.

As mentioned in the previous paragraphs, Brennand et al. (2011) were among the first to assess the effects of AP and SGA such as clozapine, olanzapine, risperidone, thioridazine, and loxapine. They exposed the iPSC-derived neurons to AP or SGA for 3 weeks, showing no effect on the phenotype, with exception of 10 μM loxapine, that was able to partially revert the impaired neuronal connectivity. However, the *in vitro* concentration probably was too high to explain a possible clinical effect (Chakrabarti et al., 2007). This lack of effects on cellular structural parameters and neuroplasticity by most AP and SGA was somewhat expected, since antipsychotics are considered to deliver a symptomatic treatment rather than a disease-modifying treatment. The reversal of functional synaptic deficits in iPSC-derived neurons from members of a family carrying DISC1 mutations previously discussed (Wen et al., 2014), was reported with incubation with the PDE4 inhibitor rolipram, a pharmacologic agent known to affect cAMP/cGMP signaling. Interestingly, increased PDE4 expression was reported by loss of function of DISC1 or ATF4 (Soda et al., 2013), supporting a rationale for pharmacological PDE4 inhibition. One exploratory study (Nakazawa et al., 2017) included iPSC-derived neurons from a pair of monozygotic twins with diagnosis of treatment-resistant SCZ and different response to clozapine. The authors found that the neuronal cultures from the iPSC-derived neurons of the clozapine-responder tween when exposed to 1 μM clozapine showed differential higher expression levels of 167 genes and lower expression levels of 95 genes, when compared to the RNA profile obtained from the clozapine-non-responder tween. Since several overexpressed genes are part of the functional group of homophilic cell adhesion molecules, the authors concluded that response to the clozapine treatment may be related to an enhancement of cell adhesion gene expression relevant for neuronal plasticity that are defective in non-responders.

More recently, evidence of a reversible dysfunctional bioenergetic signature based on profiling acyl-carnitines and mitochondrial functions was observed in subjects with SCZ (Cao et al., 2019). This study corroborates the results obtained in iPSC-derived cortical interneurons from 9 donors with SCZ, showing a consistent mitochondrial bioenergetic deficit that was reverted by *in vitro* treatment with acyl-l-carnitine (Ni et al., 2019). No information is currently available on DA neurons. These results are in line with cortical dysfunctional metabolic activities observed in subjects with SCZ (Duarte and Xin, 2019). Intriguingly, prefrontal hypometabolism has been related to NS (Wolkin et al., 1992) in the same cortical regions affected by reduced DAergic input.

Among the pharmacological models of psychosis used in humans, exposure to low dose ketamine and to psychostimulant, such as amphetamine, cocaine or methamphetamine, have been considered with interest since they mimic several aspects of the psychotic symptoms in SCZ.

Ketamine, a dissociative anesthetic that blocks central NMDA-R, produces transient increases of DA and acute psychotomimetic effects, including NS (Pomarol-Clotet et al., 2006) and it was used to explore the hypo-glutamatergic and DA cortical hypotheses of SCZ (Krystal et al., 1994; Pomarol-Clotet et al., 2006; Thiebes et al., 2017). Ketamine was recently tested on iPSC-derived DA neurons from healthy donors, resulting in increased dendritic arborization dependent upon AKT-mTOR pathway activation that requires a viable D3 receptor DA neurotransmission (Cavalleri et al., 2018). These effects remind the Akt-mTOR dysfunction reported in postmortem SCZ brain (Ryskalin et al., 2018). Similar effects, also mediated by a D3 receptor-dependent Akt-mTOR pathway, were observed in human and mouse DA neurons exposed to direct D3 receptor-preferential DA agonists, such as pramipexole or ropinirole (Collo et al., 2018) or in mouse primary DA neurons exposed to psychostimulants or D3 receptor-preferential DA agonists (Collo et al., 2012). Intriguingly, the effects of ketamine, amphetamine and of D3 receptor-preferential DA agonists were prevented by DA-D3 receptor blockers, supporting a possible mechanistic interpretation of the clinical effects on NS produced by D3-prevalent partial agonist AP, such as cariprazine (Sokoloff and Le Foll, 2017).

## Conclusion and Perspectives

Schizophrenia is a chronic, neurodevelopmental, disabling mental disorder with a severe impact on functional outcome. It is also a heterogeneous disorder, including a various blend of positive symptoms, NS and cognitive impairment. Pharmacological treatments have proven effective mostly on positive symptoms, but have not substantially improved the functional outcome for the majority of people with SCZ, as they are not effective on dimensions which account for most of the functional impairment of these people, such as NS and cognitive impairment. The remarkable heterogeneity and complexity of the disorder poses a tremendous challenge in identifying pathophysiological mechanisms in order to find novel and targeted treatments.

Translational animal models have improved the knowledge about some pathophysiological mechanisms underlying SCZ. With regard to NS, some animal models provided a better understanding of the neurobiological mechanisms of deficit in motivation, by recapitulating critical circuit impairments that are highly conserved among mammals. Unfortunately, this circuit-based model can only partially clarify the pathophysiological mechanisms of those NS which are not related to motivation, such as blunted affect and alogia. In addition, we have to take into account that animal models have some important limitations. Indeed, rats or mice exhibit profound differences with respect to humans in brain anatomy and functions, as well as some aspects of social behavior; thus how these models reflect complex symptoms, such as NS, is debatable.

The discovery of iPSC has opened the door for new translational strategies to better characterize the pathophysiological mechanisms of SCZ. Indeed, this technique provides an opportunity to study the cellular neuropathology of specific subjects with SCZ. Neuronal cultures differentiated from iPSC share the same genetic background of their donors, ideally linking a specific cellular biology with the specific clinical condition of the donors. Studies from neurological disorders associated to single gene mutations show that iPSC-derived neurons could generate precious information about the molecular mechanisms involved in driving the clinical symptoms (Avior et al., 2016). Taking into account the documented implication of DA dysfunctions in NS, a reasonable translational approach is represented by iPSC-derived DA neurons, which could provide disease-relevant phenotypes at transcriptomic and cellular levels. To date iPSC translational research is at the beginning and much more effort is needed. However, this method has the potential to characterize the heterogeneity of the disorder by effectively implement a Precision Medicine approach (Figure 1). In a near future this method might provide a meaningful characterization of SCZ subtypes e.g., subjects with primary and persistent NS, thus fostering the development of specific treatments. The clinical characterization will be essential for the identification of subtypes (or clusters) of patients aimed to iPSC donation. A suggested approach for future application of this methodology would be to characterize specific abnormalities in iPSC-derived DA neurons from subjects with prevalent or persistent NS, and use them to test novel pharmacologic agents/AP aimed to revert these changes *in vitro*. The selected pharmacologic agents/AP will be then assessed in animal translational models to further characterize effects on motivation and incentive behavior *in vivo*. Finally, the selected agents, if safe and well tolerated, will be tested in subjects with NS, assessing changes in brain circuits engaged in motivation and measuring changes of clinical scores for NS using NS assessment instruments which overcome the limits of the PANSS (e.g., The Brief Negative Symptom Scale or the Clinical Assessment Interview for Negative Symptoms). Overall, integrating a circuit-based approach to study motivation in preclinical mammals with the information coming from human iPSC-derived cellular models should provide a strong translational paradigm for testing novel potential treatments in subjects with SCZ.

In conclusion, due to the potentiality of the iPSC method to provide meaningful data also in a limited number of subjects, the classification of subjects using both categorical and dimensional perspectives (e.g., Deficit Schizophrenia with avolition or with blunted affect) might pave the way for innovative translational studies inspired to the Precision Medicine paradigms.

## Author Contributions

All authors contributed to critically revising the content of the manuscript, and approved the final manuscript for submission to Frontiers in Neuroscience.

## Conflict of Interest

General sources of potential conflict of interest, considered unrelated to this work include the following: AM has received fees as consultant and/or advisor to Janssen Pharmaceutica NV, Gedeon Bulgaria, Pierre Fabre, and Lundbeck. SG has been a consultant and/or advisor to or has received honoraria or grants from: Millennium Pharmaceuticals, Innova Pharma - Recordati Group, Janssen Pharmaceutica NV, Sunovion Pharmaceuticals, Janssen-Cilag Polska Sp. z o.o., Gedeon Richter-Recordati, Pierre Fabre, Otsuka, and Angelini. EM was employed by the company Alfasigma Schweiz.
