# Corrigendum: Negative Symptoms of Schizophrenia and Dopaminergic Transmission: Translational Models and Perspectives Opened by iPSC Techniques

**DOI:** 10.3389/fnins.2021.729082

**Published:** 2021-07-15

**Authors:** Ginetta Collo, Armida Mucci, Giulia M. Giordano, Emilio Merlo Pich, Silvana Galderisi

**Affiliations:** ^1^Department of Molecular and Translational Medicine, University of Brescia, Brescia, Italy; ^2^Department of Psychiatry, University of Campania “Luigi Vanvitelli”, Naples, Italy; ^3^Research & Development, Alfasigma Schweiz, Zofingen, Switzerland; ^4^Department of Brain Sciences, Imperial College London, London, United Kingdom

**Keywords:** schizophrenia, negative symptoms, dopamine, animal models, iPSC

In the original article, we neglected to include an Acknowledgments section. The added Acknowledgements section is below:

We would like to thank the “VAnviteLli pEr la RicErca: VALERE” program of the University of Campania Luigi Vanvitelli that assigns contributions for the diffusion of open access research products.

The authors apologize for this error and state that this does not change the scientific conclusions of the article in any way. The original article has been updated.

